# Diet-Induced Obese Mice and Leptin-Deficient *Lep^ob/ob^* Mice Exhibit Increased Circulating GIP Levels Produced by Different Mechanisms

**DOI:** 10.3390/ijms20184448

**Published:** 2019-09-10

**Authors:** Eunyoung Lee, Emily L. Miedzybrodzka, Xilin Zhang, Ryo Hatano, Junki Miyamoto, Ikuo Kimura, Kosuke Fujimoto, Satoshi Uematsu, Sergio Rodriguez-Cuenca, Antonio Vidal-Puig, Fiona M. Gribble, Frank Reimann, Takashi Miki

**Affiliations:** 1 Department of Medical Physiology, Chiba University, Graduate School of Medicine, Chiba 260-8670, Japan (E.L.) (X.Z.) (R.H.); 2 Metabolic Research Laboratories, Wellcome Trust MRC Institute of Metabolic Science, University of Cambridge, Addenbrooke’s Hospital, Cambridge CB2 0QQ, UK (E.L.M.) (S.R.-C.) (A.V.-P.) (F.M.G.) (F.R.); 3 Department of Applied Biological Science, Graduate School of Agriculture, Tokyo University of Agriculture and Technology, Fuchu 183-8509, Japan (J.M.) (I.K.); 4 Department of Immunology and Genomics, Osaka City University School of Medicine, Osaka 545-8585, Japan (K.F.) (S.U.); 5 Division of Innate Immune Regulation, International Research and Development Center for Mucosal Vaccines, The Institute of Medical Science, The University of Tokyo, Minato-ku 108-8639, Japan

**Keywords:** glucose-dependent insulinotropic polypeptide (GIP), high-fat diet, diet-induced obese (DIO) mice, leptin, *Lep^ob/ob^*, interleukin-1 receptor antagonist (IL-1Ra)

## Abstract

As glucose-dependent insulinotropic polypeptide (GIP) possesses pro-adipogenic action, the suppression of the GIP hypersecretion seen in obesity might represent a novel therapeutic approach to the treatment of obesity. However, the mechanism of GIP hypersecretion remains largely unknown. In the present study, we investigated GIP secretion in two mouse models of obesity: High-fat diet-induced obese (DIO) mice and leptin-deficient *Lep^ob/ob^* mice. In DIO mice, plasma GIP was increased along with an increase in GIP mRNA expression in the lower small intestine. Despite the robust alteration in the gut microbiome in DIO mice, co-administration of maltose and the α-glucosidase inhibitor (α-GI) miglitol induced the microbiome-mediated suppression of GIP secretion. The plasma GIP levels of *Lep^ob/ob^* mice were also elevated and were suppressed by fat transplantation. The GIP mRNA expression in fat tissue was not increased in *Lep^ob/ob^* mice, while the expression of an interleukin-1 receptor antagonist (IL-1Ra) was increased. Fat transplantation suppressed the expression of IL-1Ra. The plasma IL-1Ra levels were positively correlated with the plasma GIP levels. Accordingly, although circulating GIP levels are increased in both DIO and *Lep^ob/ob^* mice, the underlying mechanisms differ, and the anti-obesity actions of α-GIs and leptin sensitizers may be mediated partly by the suppression of GIP secretion.

## 1. Introduction

Glucose-dependent insulinotropic polypeptide (GIP), as well as glucagon-like peptide 1 (GLP-1), are the principal incretins that potentiate insulin secretion from pancreatic β cells and reduce postprandial hyperglycemia [1]. GIP and GLP-1 are secreted from enteroendocrine K cells and L cells, respectively, and act on the target cells through binding to their specific receptors, GIPR and GLP-1R. GIPR is shown to be expressed not only in pancreatic β cells, but also in other tissues in which GIP exerts pleiotropic actions [2]. Notably, GIPR is expressed in adipose tissues and GIP has been reported to increase fat accumulation in white adipose tissues [3]. A connection between obesity and GIP has been reported: Plasma GIP levels in obese humans are increased [4], and GIPR knockout mice are resistant to high-fat diet (HFD)-induced obesity [5]. In addition, HFD feeding was found to increase mRNA expression of GIP [6] and to induce obesity through the binding of meal-derived free fatty acids to its binding protein, fatty acid binding protein 5 (FABP5) [7]. They also found that induction of HFD-induced obesity requires GIP function using knockout mice of GIP and/or FABP5 [7]. Accordingly, GIP is considered to act as a pro-adipogenic hormone that exacerbates obesity by forming a vicious circle of obesity and hypersecretion of GIP [8]. Indeed, several GIP receptor antagonists are under development with the goal of reducing body weight in humans [9,10]. However, in contrast to the well-defined action of GIP on adipose tissues, the regulatory mechanism of GIP secretion in the context of obesity has not been fully clarified.

We previously reported that oral administration of the disaccharide maltose in addition to the α-glucosidase inhibitor (α-GI) miglitol potentiates GLP-1 secretion [11] while suppressing GIP secretion [12]. GIP secretion was reduced by the co-administration of maltose and miglitol (maltose/miglitol) in wild-type (WT) mice under standard SPF (specific pathogen-free) conditions, but not in germ-free WT mice or in mice lacking the short-chain fatty acid (SCFA) receptor GPR41 (FFAR3). Therefore, the mechanism of the GIP suppression appeared to be mediated by the activation of GPR41 by SCFAs that are produced from maltose by the microbiome [13]. Interestingly, GPR41 was found to be expressed in the K cells of the lower small intestine, but not in those of the upper intestine [14]. Therefore, contrary to the current consensus that GIP is mainly released from the upper small intestine, the apparent reduction in GIP secretion by maltose/miglitol administration suggests that a considerable amount of GIP is released from the K cells in the lower small intestine. Thus, we hypothesized that if the increase in GIP mRNA occurs only in the upper small intestine in DIO mice, α-GI would be no longer effective in suppressing GIP secretion. If this were the case, α-GI might not be effective in ameliorating GIP hypersecretion in patients with excessive fat intake-associated obesity. In addition to GPR41, other fatty acid receptors have been shown to be differentially expressed in K cells in the upper and lower small intestine [14]. Recently, the regulatory mechanism of serotonin secretion from enterochromaffin (EC) cells has also been reported to differ depending on the position of EC cells in the intestine [15]. Thus, the cellular phenotype of K cells must be better evaluated for each location of the small intestine. In the present study, we examined the HFD-induced alterations in the gut microbiome, SCFA levels in the portal vein, GIP expression in the gut, and GIP secretion in diet-induced obese (DIO) mice. In addition, in order to discriminate the direct action of HFD on K cells in the gut lumen and the contribution of the consequent obesity to circulating GIP levels, we also examined the regulatory mechanism of GIP secretion in *Lep^ob/ob^* mice, a murine obese model that develops obesity under normal chow diet (ND).

## 2. Results

### 2.1. HFD Feeding Increased Plasma GIP Concentration along with an Increase of K Cell Number and GIP mRNA Expression in the Lower Small Intestine

**Figure 1:**
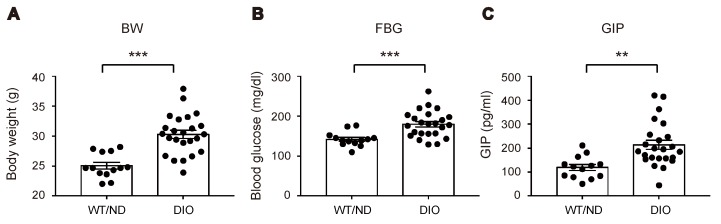
Effect of high-fat diet (HFD) on body weight, blood glucose levels, and plasma glucose-dependent insulinotropic polypeptide (GIP) levels. Body weight (**A**), blood glucose levels (**B**), and plasma GIP levels (**C**) were measured in wild-type (WT) mice fed normal chow diet (ND) (WT/ND) or HFD (diet-induced obese, DIO) for four weeks, then fasted overnight before sampling. Each data point is plotted as a circle. Bars depict mean ± SEM. ** *p* < 0.01, *** *p* < 0.001.

**Figure 2:**
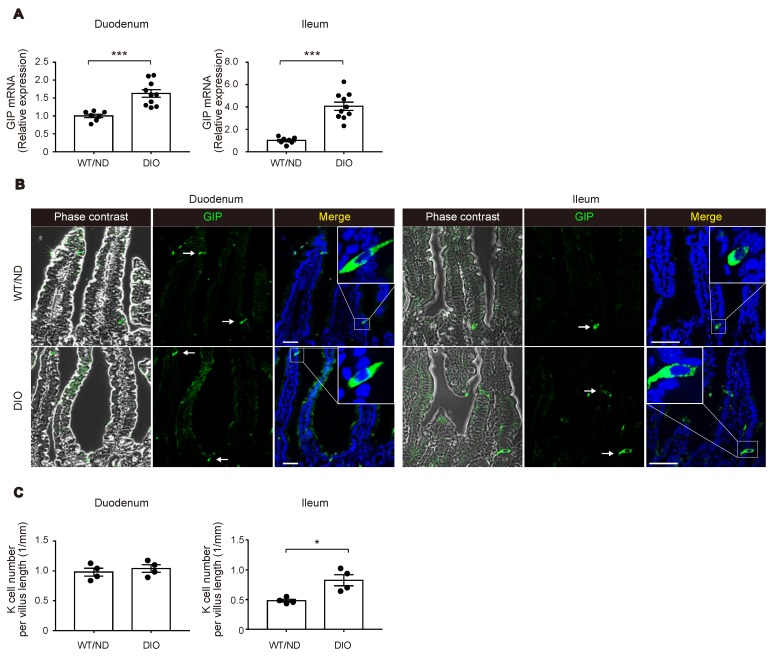
Changes in GIP expression and K cell number by HFD. (**A**) Messenger RNA expression of GIP in the duodenum (left) and the terminal ileum (right) was examined in WT/ND and DIO mice by quantitative real-time polymerase chain reaction (qPCR) analysis. (**B**,**C**) Immuno-histochemical images of GIP-positive K cells (**B**) and quantification of K cell number (**C**). (**A**,**C**) Each data point is plotted as a circle. Bars depict mean ± SEM. * *p* < 0.05, *** *p* < 0.001. (**B**) Bars indicate 50 μm.

We first measured plasma levels of GIP in wild-type (WT) mice fed on ND or HFD (WT/ND or DIO, respectively) for four weeks. HFD feeding significantly increased plasma GIP levels, as well as body weight and fasting blood glucose levels in DIO mice (Figure 1A–C). Next, we examined the HFD-induced increase of GIP mRNA expression in both the duodenum and the terminal ileum (Figure 2A). GIP expression was modestly (~1.5-fold) increased by HFD in the duodenum, while it was robustly (~4-fold) increased in the terminal ileum. Considering that an increase in the number of K cells might contribute to the increase in the amount of GIP mRNA, we also quantified K cell number (Figure 2B,C). Interestingly, the K cell number was not changed by HFD feeding in the duodenum, the region in which K cells occur most abundantly in the gut, but it was significantly increased in the terminal ileum.

### 2.2. HFD Feeding Altered the Qualitative Composition of the Gut Microbiome, but Did not Affect the Maltose/Miglitol-Induced Increase of Plasma SCFAs or Maltose/Miglitol-Induced Suppression of Plasma GIP

**Figure 3:**
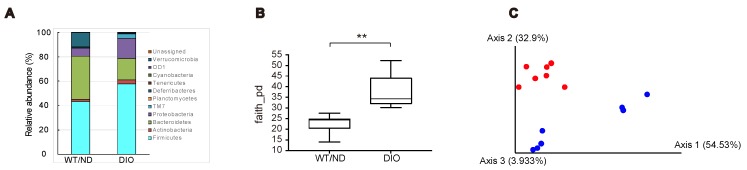
Gut microbial compositions and diversities in DIO and WT/ND mice four weeks after HFD or ND administration. (**A**) Gut microbial compositions based on the relative abundance of OTUs (operational taxonomic units), the units determined by classifying groups of closely related microbiome, at the phylum level from DIO (*n* = 7) and WT/ND mice (*n* = 7). (**B**) Bacterial alpha diversities for the fecal microbial community using Faith’s phylogenetic alpha diversity index. The *p* value was calculated using the Kruskal-Wallis test. ** *p* < 0.01. (**C**) PCoA (principal coordinate analysis) on the weighted UniFrac distance matrices for the microbial community of DIO mice (blue) and WT/ND (red).

We previously showed that SCFAs produced by the microbiome are essential for inhibiting GIP secretion by maltose/miglitol [12]. However, HFD feeding has been reported to change the microbiome [16] and SCFA production in the gut [17]. Therefore, we first examined the changes in the microbiome after four-week HFD feeding. The relative abundance of gut microbes at the phylum level showed that the gut microbial composition was changed in DIO mice (Figure 3A). Notably, the relative abundance of *Firmicutes*, *Actinobacteria*, *Proteobacteria,* and *TM7* were increased, whereas that of *Bacteroidetes* and *Verrcomicrobia* were decreased by the HFD. The index of bacterial alpha diversity (Faith’s phylogenetic alpha diversity [18], an index reflecting how diverse is the microbiome residing in the gut), was significantly higher in DIO mice compared with WT/ND mice (Figure 3B). In addition, a Principal coordinate analysis (PCoA, a statistical method to explore similarities within a dataset) for the weighted UniFrac distance matrices [19] showed that the gut microbial community of DIO mice was clearly distinguished from that of WT/ND mice (Figure 3C). Collectively, these findings indicate that HFD induces qualitative changes in the intestinal microbiome. 

**Figure 4:**
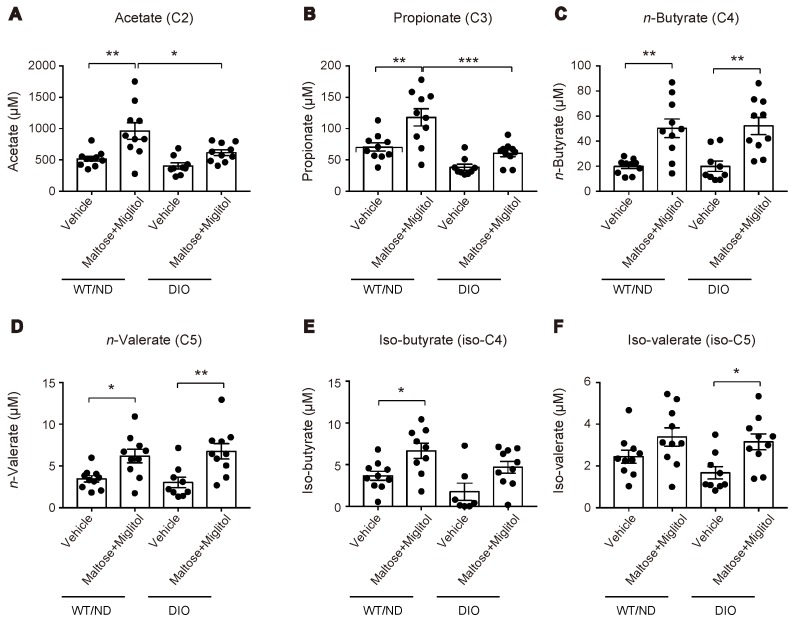
Plasma short-chain fatty acid (SCFA) levels in the portal vein after maltose/miglitol administration. (**A**–**F**) Plasma SCFAs (acetate (**A**), propionate (**B**), *n*-butyrate (**C**), *n*-valerate (**D**), iso-butyrate (**E**), and iso-valerate (**F**)) in the portal vein at 30 min after oral administration of maltose + miglitol or vehicle to DIO and WT/ND mice. The data of the control mice are the same as those published previously by Lee et al. [12] and are presented for comparison with those of DIO mice. Bars depict mean ± SEM. * *p* < 0.05, ** *p* < 0.01, *** *p* < 0.001.

**Figure 5:**
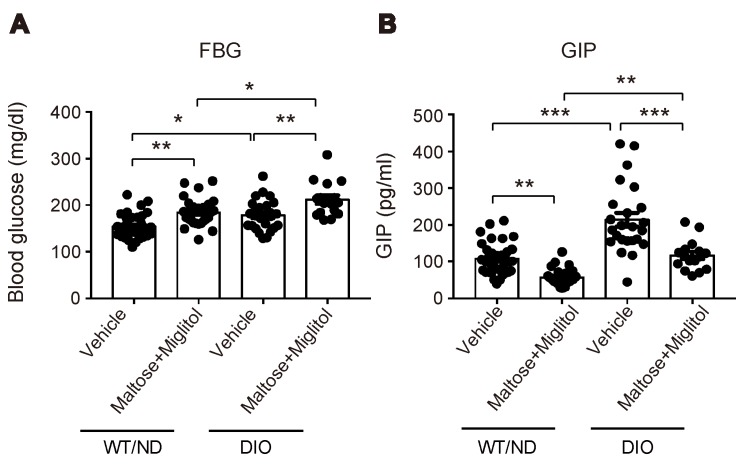
Blood glucose and plasma GIP levels in the portal vein after maltose + miglitol administration. (**A**,**B**) Levels of blood glucose (**A**) and plasma GIP (**B**) in the portal vein at 30 min after oral administration of maltose + miglitol or vehicle to DIO and WT/ND mice (*n* = 16–34 for each group). The data of the control mice are the same as those published previously by Lee et al. [12] and are presented for comparison with those of DIO mice. Bars depict mean ± SEM. * *p* < 0.05, ** *p* < 0.01, *** *p* < 0.001.

HFD feeding also changed the variations of the plasma SCFA levels after maltose/miglitol administration in the portal vein (Figure 4). In spite of the alteration in the gut microbiome by HFD feeding, the basal levels (i.e., vehicle) of plasma SCFAs in the portal vein were not significantly altered. By contrast, plasma levels of acetate (C_2_) and propionate (C_3_) in maltose/miglitol-treated DIO mice were significantly lower than those in WT/ND mice. However, plasma levels of *n*-butyrate (C_4_), *n*-valerate (C_5_), iso-butyrate (C_4_), and iso-valerate (C_5_) in maltose/miglitol-treated DIO mice were not significantly different from those of WT/ND mice, indicating that the production of these SCFAs from maltose is not impaired in DIO mice. As butyrate and valerate are potent agonists of GPR41 [20,21], we anticipated that GIP secretion would be suppressed by maltose/miglitol administration in DIO mice, as it is in WT/ND mice. In fact, the plasma GIP levels in DIO mice were significantly higher than those in WT/ND mice and were significantly suppressed by maltose/miglitol administration (Figure 5). Taken together with the lack of expression of GPR41 in the upper small intestine [14], our results suggest that GIP released from the lower small intestine plays a role in increasing plasma GIP levels by HFD while αGI remains effective in ameliorating hypersecretion of GIP under HFD.

### 2.3. Transplantation of Wild-Type Subcutaneous Fat to Lep^ob/ob^ Mice Ameliorated Obesity, Glucose Intolerance, and Insulin Resistance

**Figure 6:**
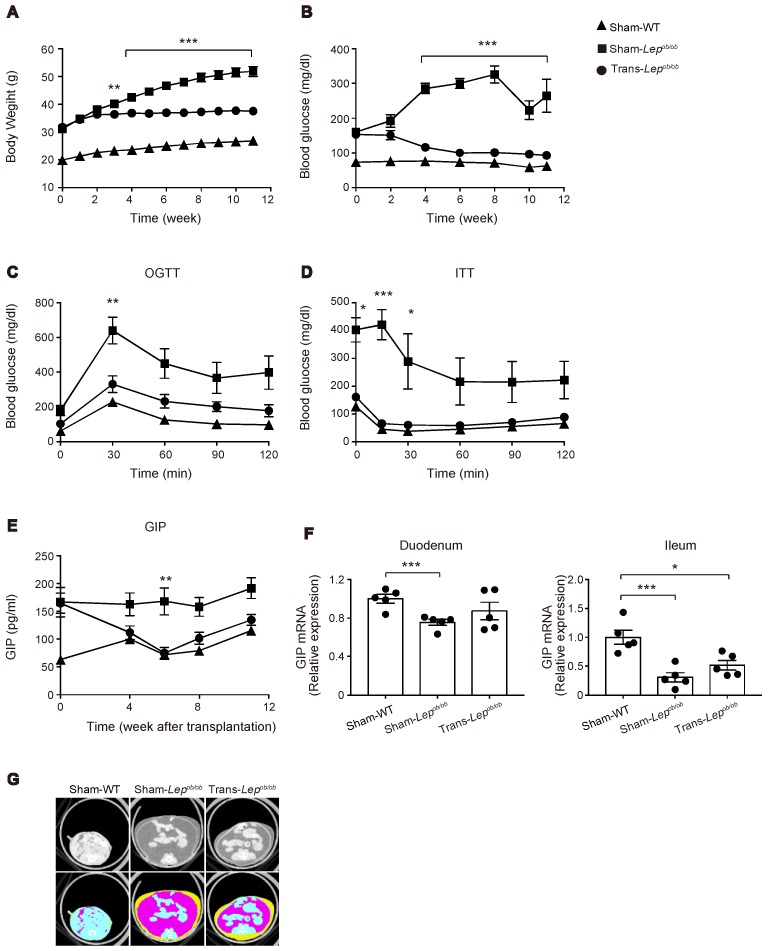
Effect of fat transplantation to *Lep^ob/ob^* mice on body weight, glucose metabolism, plasma GIP levels, GIP expression in the gut, and adiposity. (**A**–**G**) Body weight (**A**), blood glucose levels (**B**), oral glucose tolerance test (**C**), insulin tolerance test (**D**), plasma GIP levels (**E**), mRNA expression of GIP in the gut (**F**), and CT images (**G**) of Trans- *Lep^ob/ob^*, Sham- *Lep^ob/ob^*, and Sham-WT. (**A**–**G**) Results of Trans-*Lep^ob/ob^* (circles), Sham-*Lep^ob/ob^* (squares), and Sham-WT (triangles) are shown. Data are expressed as mean ± SEM. (**A**–**D**,**E**) *n* = 9–10; (**F**) *n* = 5. * *p* < 0.05, ** *p* < 0.01, *** *p* < 0.001. (**G**) Representative CT images at the level of lower end of right kidney. Plain images (upper) and colored images (lower) of visceral (pink) and subcutaneous fat (yellow).

Although the DIO mouse is useful for analyzing the direct action of HFD on the gut, HFD feeding also induces obesity, which itself may contribute to hypersecretion of GIP. We therefore evaluated GIP secretion in leptin-deficient *Lep^ob/ob^* mice, which develop severe obesity under the normal chow diet. Although *Lep^ob/ob^* mice have been reported to show increased GIP secretion [22,23], the mechanism remains unknown. In order to investigate the role of leptin signaling in GIP secretion, we transplanted subcutaneous adipose tissues surgically removed from WT/ND mice into *Lep^ob/ob^* mice. We monitored plasma GIP levels in fat-transplanted mice (Trans-*Lep^ob/ob^*), sham-operated *Lep^ob/ob^* mice (Sham-*Lep^ob/ob^*), and sham-operated wild-type mice (Sham-WT). As reported previously [24], fat transplantation to *Lep^ob/ob^* mice was found to markedly blunt the increase in body weight of *Lep^ob/ob^* mice (Figure 6A) and to significantly lower the fasting plasma glucose level (Figure 6B). We also evaluated glucose tolerance and insulin sensitivity by oral glucose tolerance test (at nine weeks after fat transplantation, Figure 6C) and intraperitoneal insulin tolerance test (at 10 weeks after fat transplantation, Figure 6D), and found that glucose tolerance and insulin sensitivity were markedly improved by fat transplantation.

### 2.4. The Plasma GIP Concentration Was Increased in Lep^ob/ob^ Mice, but Was Significantly Suppressed by Fat Transplantation 

In accord with previous literature [23], the plasma GIP levels in Sham-*Lep^ob/ob^* were significantly higher than those in Sham-WT. In addition, we found that fat transplantation to *Lep^ob/ob^* mice significantly lowered the plasma GIP levels (Figure 6E). In order to examine the mechanism of hypersecretion of GIP in *Lep^ob/ob^* mice, we first quantified mRNA expression of GIP in the duodenum and the terminal ileum. In contrast to the increase in mRNA expression of GIP in the gut by HFD feeding, GIP mRNA expression was not increased in Sham-*Lep^ob/ob^* (Figure 6F), indicating that the observed increase of GIP secretion in *Lep^ob/ob^* mice is not attributable to an increase in GIP transcription. 

Notably, although fat transplantation to *Lep^ob/ob^* mice markedly reduced the increase in their body weight, their body adiposity was still much higher than that of Sham-WT (Figure 6G). Nevertheless, plasma GIP levels of Trans-*Lep^ob/ob^* were decreased to a level comparable to those of Sham-WT at six weeks after fat transplantation, suggesting that a factor other than reduced adiposity, such as cessation of hyperphagia, slowdown in the increasing-rate of adiposity, or hormonal factor(s) released from the transplant, may contribute to the downregulation of GIP secretion in Trans-*Lep^ob/ob^* mice.

### 2.5. Expressions of Several Pro-Inflammatory Cytokines Were Increased in Epididymal Fat Tissues of Lep^ob/ob^ Mice, which Were Partially Ameliorated by Fat Transplantation 

Despite the reduction in body weight gain by fat transplantation, Trans-*Lep^ob/ob^* still had higher adiposity when compared with Sham-WT. By contrast, the amount of fat transplant of Trans-*Lep^ob/ob^* was relatively small (~0.9 g on sampling). We therefore hypothesized that the endogenous, leptin-deficient fat tissues had been affected by the transplanted fat through the action of leptin secreted from the transplant, which then contributed to amelioration of hypersecretion of GIP.

**Figure 7:**
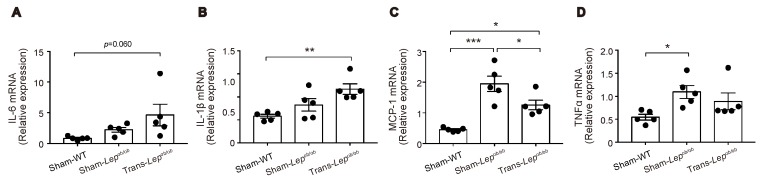
Effect of fat transplantation to *Lep^ob/ob^* mice on mRNA expressions of inflammation-related genes in epididymal fat tissues. (**A**–**D**) Messenger RNA expressions of IL-6, IL-1β, MCP-1, and TNFα in Trans- *Lep^ob/ob^*, Sham- *Lep^ob/ob^*, and Sham-WT. Data are expressed as mean ± SEM. * *p* < 0.05, ** *p* < 0.01, *** *p* < 0.001.

Adipose tissues of *Lep^ob/ob^* mice are known to exhibit enhanced inflammation associated with macrophage infiltration [25]. We therefore examined the mRNA expressions of these genes in epididymal fat tissues (Figure 7A–D). Messenger RNA expression levels of interleukin-6 (IL-6) and interleukin-1β (IL-1β) tended to be higher in Sham-*Lep^ob/ob^* than in Sham-WT, and fat transplantation increased rather than suppressed the expression of these mRNAs, suggesting that the reduction of IL-6 or IL-1β from epididymal fat tissues is unlikely to have contributed to the decrease in GIP secretion by fat transplantation. In contrast, mRNA expression levels of monocyte chemoattractant protein-1 (MCP-1) and tumor necrosis factor-α (TNFα) were significantly higher in Sham-*Lep^ob/ob^* than those in Sham-WT and tended to be suppressed by fat transplantation, suggesting that these pro-inflammatory cytokines are candidates for participation in the observed changes in GIP secretion.

### 2.6. Messenger RNA and Protein Levels of Interleukin-1 Receptor Antagonist (IL-1Ra) in Lep^ob/ob^ Mice Were Elevated, but Were Suppressed by Fat Transplantation, and Its Protein Levels Were Positively Correlated with Plasma GIP Concentrations

**Figure 8:**
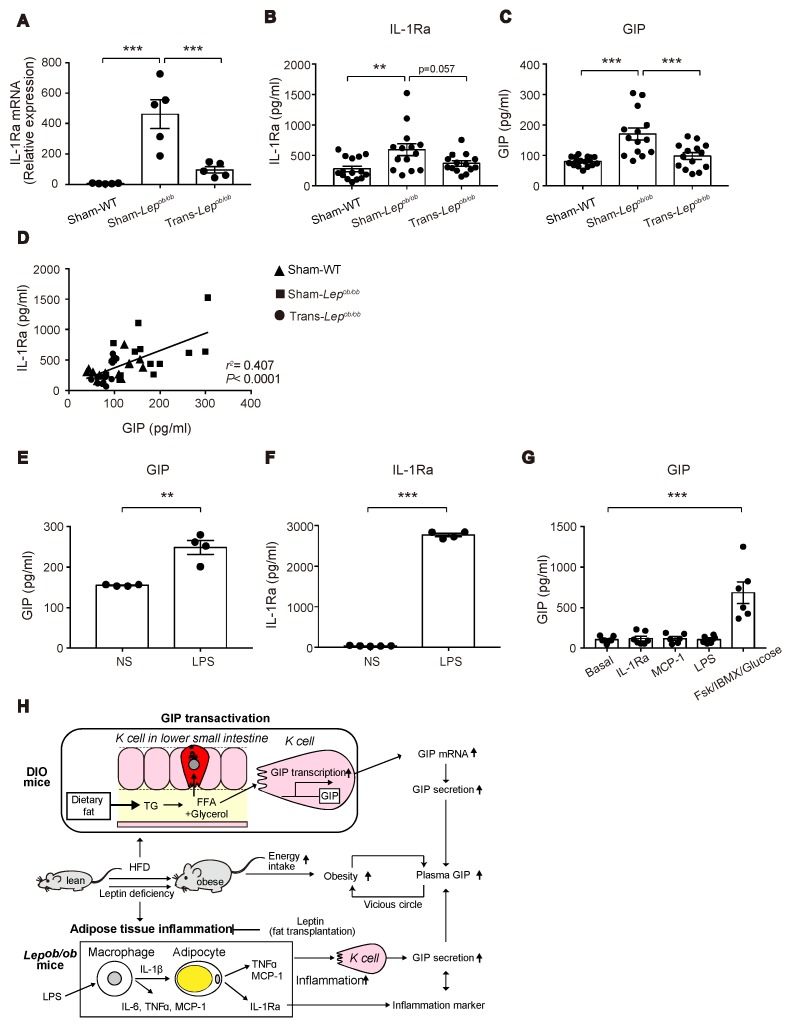
Effect of fat transplantation and LPS administration on GIP and IL-1Ra and a model for the mechanism of hypersecretion of GIP in obesity. (**A**–**C**) IL-1Ra mRNA expression in epididymal fat and plasma levels of IL-1Ra (**B**) and GIP (**C**) in Trans-*Lep^ob/ob^*, Sham-*Lep^ob/ob^*, and Sham-WT. (**D**) Scatterplots of plasma levels of IL-1Ra and GIP of Trans-*Lep^ob/ob^* (circles), Sham-*Lep^ob/ob^* (squares), and Sham-WT (triangles). The dotted line depicts the regression line for all data (*r^2^* = 0.407, *p* < 0.0001). (**E**,**F**) Plasma levels of GIP (**E**) and IL-1Ra (**F**) at 3 h after LPS administration to WT mice. (**G**) GIP secretion from mouse small intestinal organoids after 2 hr stimulation with MCP-1, IL-1Ra, LPS or forskolin/IBMX/10 mM glucose (FSK/IBMX/Glucose). (**A**–**C**,**E**–**G**) Data are expressed as mean ± SEM. ** *p* < 0.01, *** *p* < 0.001. (**H**) A model of hypersecretion of GIP in obesity. See the text in detail.

We then examined mRNA and protein levels of IL-1Ra, a cytokine released from inflamed adipose tissues. Interestingly, mRNA expression levels of *Il-1Ra* were higher in Sham-*Lep^ob/ob^* than in Sham-WT and were suppressed by fat transplantation (Figure 8A), suggesting that increased expression of *Il-1Ra* in adipose tissues may have some relevance to the increase of GIP in Sham-*Lep^ob/ob^*. 

We therefore measured the circulating levels of IL-1Ra in Trans-*Lep^ob/ob^*, Sham-*Lep^ob/ob^*, and Sham-WT at six weeks after the transplantation, the time point at which the decrease in plasma GIP levels was the greatest (Figure 8B). Notably, the plasma levels of IL-1Ra in Sham-*Lep^ob/ob^* were significantly higher than those of Sham-WT and tended (*p* = 0.057) to be suppressed by fat transplantation (Figure 8C). In addition, there was a positive correlation between plasma IL-1Ra levels and plasma GIP levels (*r^2^* = 0.407, *p* < 0.0001) (Figure 8D), suggesting a link between plasma IL-1Ra levels and GIP secretion. 

### 2.7. Lipopolysaccharide (LPS) Administration to Mice Increased Plasma Levels of GIP and IL-1Ra

Visceral fat tissues were reported to be a major source of IL-1Ra under obesity and pro-inflammatory conditions experimentally induced by administration of LPS, phorbol myristate acetate (PMA), IL-1, and interferon-β (IFN-β) [26]. Accordingly, a positive correlation between plasma IL-1Ra and GIP may suggest *a priori* that inflammation in adipose tissues upregulates GIP secretion in *Lep^ob/ob^* mice. To test this hypothesis, we treated wild-type mice with LPS, which triggers the release of various pro-inflammatory cytokines including IL-1β, IL-6, and TNFα, and measured plasma GIP concentrations. As predicted, LPS administration increased GIP secretion (Figure 8E), which was associated with a marked increase in plasma IL-1Ra levels (Figure 8F). Accordingly, our data suggest that the inflammation in adipose tissues induced by leptin-deficiency may contribute to the upregulation of GIP secretion from K cells.

### 2.8. GIP Secretion Was Not Triggered in the Small Intestinal Organoids by LPS, MCP-1, or IL-1Ra

We then examined acute GIP secretion in vitro using small intestinal organoid-derived cultures. GIP secretion was significantly stimulated in response to forskolin plus IBMX, while there was no effect of LPS, MCP-1, or IL-1Ra on acute GIP secretion (Figure 8G). This suggests that the increased GIP secretion in *Lep^ob/ob^* mice is not mediated by the direct action of these agents on K cells.

## 3. Discussion

Circulating levels of GIP are increased in obesity in humans and rodents. However, the underlying mechanism has not been fully clarified. Shibue et al. found that HFD increased GIP secretion through the binding of meal-derived free fatty acids to FABP5 [7]. In addition, HFD has also been shown to increase GIP expression in K cells [6], shedding light on the importance of GIP transcription in the pathogenesis of GIP hypersecretion under HFD. We previously found that co-administration of the α-GI miglitol and maltose suppresses GIP secretion through the microbiome/SCFA/GPR41 pathway [12]. Since GPR41 has been shown to be expressed in the lower small intestine but not in the upper small intestine [14], the robust suppression of GIP secretion by oral maltose/miglitol administration strongly suggests that a considerable amount of GIP secretion in the fasting stage originates from the lower small intestine, in which K cells are less abundant.

In the present study, we found that GIP mRNA expression was induced to a greater extent in the lower small intestine compared to the upper small intenstine following HFD feeding, supporting our concept that physiologically relevant amount of GIP is released from K cells in the lower small intestine, even though their relative abundance is less than in the upper small intestine. The molecular mechanism underlying the robust activation of GIP release in the lower small intestine, but not in the upper small intestine, remains unknown. However, this finding suggests that the cellular properties of K cells differ considerably depending on their location in the small intestine. Messenger RNA expression of GPR40, the receptor for long-chain fatty acids, was shown to be expressed in K cells in the lower small intestine but not in those in the upper small intestine [14]. Therefore, the ingestion of HFD may be sensed by GPR40 on K cells in the lower small intestine, leading to an increase in GIP expression. Judging from the increase in K cell number in the lower small intestine following HFD feeding, it is possible that HFD feeding influences the differentiation of K cells, although the underlying mechanism remains unknown at present.

In addition, our present study suggests that α-GI may be effective in suppressing hypersecretion of GIP in human obesity. α-GIs are widely used for the treatment of diabetes mellitus. They inhibit the hydrolysis of poly- or oligo-saccharides to mono-saccharides, leading to a suppression of the postprandial rise in glycemia [27]. Currently, three compounds (acarbose, voglibose, and miglitol) are in clinical use, and all three have been found to induce body weight reduction [28,29,30]. Miglitol is known to be absorbed from gut lumen and has been reported to exert an anti-obesity effect by activating brown adipose tissues in mice [30]. By contrast, acarbose and voglibose are poorly absorbable from the gut lumen, suggesting that the body weight reduction effect of α-GIs is likely mediated by their properties as inhibitors of α-glucosidase. Since acarbose and miglitol were both shown to suppress GIP secretion in response to co-administration of maltose in mice [12], GIP suppression by α-GIs may contribute, at least in part, to the anti-obesity effect in obese human subjects with diabetes mellitus.

Although DIO mice are a useful model for studying the direct effect of HFD on GIP secretion, HFD feeding induces obesity. Thus, the increase in GIP secretion in DIO mice might be influenced by the increase in adiposity, as well as by direct stimulation of K cells in the gut lumen. To distinguish these two factors, we utilized *Lep^ob/ob^* mice, which develop severe obesity under normal-fat chow diet feeding. *Lep^ob/ob^* mice are obese, but are also known to develop other conditions, including hyperglycemia, insulin resistance, and infertility. Because the infertility of *Lep^ob/ob^* mice cannot be restored by body weight reduction induced by food restriction [31], the leptin-signaling defect itself is considered responsible for the particular phenotype independent from the consequent obesity. We therefore hypothesized that defective leptin-signaling may have contributed, at least partially, to the increased GIP secretion. In accord with the previous report [23], plasma GIP levels were found to be increased in *Lep^ob/ob^* mice. In addition, the elevation in plasma GIP was ameliorated by fat transplantation. Unlike the case of DIO mice, expression of GIP in the gut of *Lep^ob/ob^* mice was not increased, suggesting the involvement of a distinct mechanism. Adipose tissues of *Lep^ob/ob^* mice are known to exhibit enhanced inflammation associated with macrophage infiltration [25]. In adipose tissues in obesity, expressions of pro-inflammatory cytokines such as MCP-1, IL-6, TNFα, and IL-1β in macrophages and/or adipocytes have been reported to increase [32,33]. We then focused on the possible involvement of enhanced inflammation in the adipose tissues of *Lep^ob/ob^* mice, as pro-inflammatory conditions in fat tissues were previously reported to contribute to insulin resistance and perturbation of glucose metabolism in *Lep^ob/ob^* mice [25,34]. Notably, in accord with our present study, the previous literature found that LPS induced GIP secretion [35]. They also showed that IL-1 signaling is required for the GIP secretion by LPS using IL-1R knockout mice.

IL-1 is a potent accelerator of chronic inflammation in adipose tissues that plays a central role in the pathogenesis of insulin resistance [36,37]. IL-1Ra, as well as IL-1β, are members of the IL-1 family that binds to IL-1 receptor. Although IL-1β is a potent accelerator of chronic inflammation in adipose tissues that plays a central role in pathogenesis of insulin resistance [36,37], IL-1Ra exerts an anti-inflammatory effect by blocking the action of IL-1α and IL-1β on the IL-1 receptor [34]. In fact, IL-1Ra knockout mice have been reported to exhibit increased inflammatory status and develop spontaneous autoimmune arthritis in an IL-17- and T-cell dependent manner. Nevertheless, IL-1Ra expression is potently induced by various pro-inflammatory stimuli including LPS, IL-1α, IL-1β, and IFN-β in primary cultures of human adipose tissue [38].

In addition to the classical inflammatory conditions, the expression of IL-1Ra is reported to be increased in adipose tissues in obesity [39]. Both pro-inflammatory cytokines mentioned above and the increase in IL-1Ra expression by leptin-deficiency have been reported to be prominent in epididymal fat tissue, a typical visceral fat depot that contributes more greatly to the increase in circulating levels of inflammatory cytokines than subcutaneous fat depots [37]. Accordingly, elevation of plasma IL-1Ra in obesity is considered to reflect the enhanced inflammation in adipocytes and insulin resistance. In accord with this, human studies in non-diabetic African-Americans clarified that elevated circulating levels of IL-1Ra are associated with insulin resistance [40]. Furthermore, a large cohort study of non-diabetic subjects revealed that IL-1Ra is a potent biomarker for insulin resistance and risk of type 2 diabetes mellitus [41]. Thus, increased IL-1Ra protein in the plasma may not participate in inducing inflammation, but may rather serve as a biomarker that reflects the inflammatory status of the adipose tissues.

Accordingly, the observed suppression of the elevated plasma IL-1Ra levels of *Lep^ob/ob^* mice by fat transplantation suggests that leptin released from the transplant ameliorated inflammation of the endogenous visceral fat tissues, leading to the decline in plasma IL-1Ra and GIP secretion from K cells. In order to clarify whether we could identify any humoral factor(s) from the inflamed adipocytes that directly stimulates K cells, we examined GIP secretion from mouse small intestinal organoids. However, our present results do not support the possibility that acute GIP secretion is directly induced by MCP-1 or IL-1Ra. Nevertheless, this does not exclude the possibility that other cytokines from adipocytes or macrophages may trigger GIP secretion. In addition, neuronal regulation might be involved in LPS-induced GIP secretion in vivo. Identifying why LPS acutely induces GIP secretion in vivo, but not in vitro, is key to understanding the mechanism of hypersecretion of GIP under inflammatory conditions.

Based on our results of the correlation between IL-1Ra and GIP and the GIP secretion by LPS, we concluded that elevated fat inflammation in *Lep^ob/ob^* mice increases GIP secretion from K cells. 

Obesity is characterized by an increase in adiposity, but its pathophysiology contains various alterations in many tissues. The excess intake of energy from food is a primary cause for the development of obesity both in rodents and humans, and leptin plays a central role in this regulation. In *Lep^ob/ob^* mice, genetic disruption of the leptin gene leads to the hyperphagia, resulting in a marked increase in adiposity. In addition to the alteration in feeding behavior, leptin-deficiency evokes inflammatory conditions in peripheral tissues, including the liver, skeletal muscle, and fat tissues. Inflammatory changes in visceral fat tissues of *Lep^ob/ob^* mice are shown to contribute to the development of insulin resistance. In accord with this, our present data suggest that leptin replenishment by fat transplantation to *Lep^ob/ob^* mice ameliorated hypersecretion of GIP, as well as insulin resistance. In both rodents and humans, leptin resistance plays a central role in the development of obesity, and leptin sensitizers have drawn much attention with the aim to ameliorate obesity and insulin resistance. In fact, the leptin sensitizers, such as celastrol and withaferin, have been reported to show both anti-diabetic and anti-obesity effects in mice [42,43]. Considering that leptin replenishment by fat transplantation to *Lep^ob/ob^* mice and administration of these leptin sensitizers to DIO mice [42,43] may have similar effect in mouse metabolic conditions, it would be of interest to examine the effect of leptin sensitizers on GIP secretion in DIO mice.

Interestingly, the treatment of human adipocytes with GIP has been reported to induce mRNA expression of pro-inflammatory cytokines including IL-6, IL-1β, and IL-1Ra [44]. Thus, inflammation of adipose tissues and hypersecretion of GIP stimulate each other, resulting in a vicious circle of worsening insulin resistance and obesity. 

In conclusion, our present study shows that both DIO mice and *Lep^ob/ob^* mice exhibit elevated circulating GIP levels, which are produced through distinct mechanisms: The former involving GIP transcription, and the latter potentially involving adipose-tissue inflammation (Figure 8H).

## 4. Materials and Methods

### 4.1. Animals

Male WT mice and *Lep^ob/ob^* mice with a genetic background of C57BL/6J were used for the study. *Lep^ob/ob^* mice were purchased from Japan SLC, Inc. (Shizuoka 431-1103, Japan). The mice were maintained on normal standard chow diet (CE-2) (12.1 % kcal from fat) (CLEA Japan Inc., Tokyo, Japan) or on HFD (see below). For HFD feeding, WT mice were maintained on HFD (D12492) (60.0% kcal from fat) (Research Diets Inc., New Brunswick, NJ, USA) for four weeks, starting at eight weeks of age. All animal studies were approved by the Animal Care and Use Committee of Chiba University (A28-093, A29-176, and A30-221; approved on 16 Jan. 2017, 1 Feb. 2018, and 24 Mar. 2019, respectively). The use of intestinal tissue from WT mice for organoid generation was approved by the University of Cambridge Animal Welfare and Ethical Review Board and performed under Home Office Project License PE5OF6065.

### 4.2. Experiments on DIO Mice

#### 4.2.1. Measurement of Plasma GIP, K Cell Number, and GIP Expression of DIO Mice

After four weeks of HFD feeding, DIO mice were fasted overnight and subjected to collection of samples as follows: (1) The portal vein (for measuring blood glucose and plasma GIP) and (2) the duodenum and the terminal ileum (for immunostaining of GIP and for qPCR analysis of GIP). WT/ND mice were used as controls. On the day of sampling, the mice were anesthetized with 1.5–2.0% isoflurane and laparotomized. Blood was collected from the portal vein. The mice were then euthanized by decapitation and the duodenum and the terminal ileum were cut off.

#### 4.2.2. Immuno-Histological Analysis of the Gut

Immunostaining of GIP of the gut was performed by standardized methods. The duodenum and the terminal ileum were excised, fixed with 4% paraformaldehyde, and embedded in Tissue-Tek^®^ O.C.T. Compound (Sakura Finetechnical Co., Tokyo, Japan). Cryosections were stained with GIP antibody (H-027-02) from Phoenix Pharmaceuticals, Inc. (Burlingame, CA, USA). The slides were examined under a FluoView FV10i confocal microscope (Olympus, Tokyo, Japan).

#### 4.2.3. 16S rRNA Gene Analysis

Bacterial DNA extraction from each fecal sample was performed as described previously [45]. The 16S rRNA V3–V4 region was amplified by polymerase chain reaction (PCR) and purified as described previously [46]. Each DNA amplicon library was mixed in the same amount and sequenced on the MiSeq instrument (Illumina, San Diego, CA, USA) using a MiSeq v3 Reagent kit with 15% PhiX (Illumina, San Diego, CA, USA). The 16S rRNA gene analysis was performed using QIIME2 (Version; 2018.11) (https://qiime2.org). Briefly, the sequences were subjected to primer sequence trimming, quality filtering, and paired-end read merging using the dada2 denoise-paired method (--p-trim-left-f 17 --p-trim-left-r 21 --p-trunc-len-f 275 --p-trunc-len-r 215 --p-n-threads 4) (Ref, https://www.ncbi.nlm.nih.gov/pubmed/27214047). Alpha and beta diversity analyses were performed by QUIIME diversity core-metrics-phylogenetics based on the rarefied sample sequences (–p-sampling-depth 30,939 reads). Before the taxonomic analysis, the sequences of the 16S rRNA V3-V4 region were extracted from Greengenes 13_8 99% OTUs (operational taxonomic units) and our primer sequences using the q2-feature-classifier. Then, the Naive Bayes classifier was trained using the extracted Greengenes 13_8 reference sequences and Greengenes 13_8 99% OTU taxonomy. The taxonomic composition was visualized using QUIIME taxa barplot. Faith’s phylogenetic alpha diversity estimate and PCoA (principal coordinate analysis) of the weighted UniFrac distance matrices were performed using QIIME2.

#### 4.2.4. Measurement of Portal SCFAs and GIP after Maltose/Miglitol Administration

Plasma levels of SCFAs and GIP in the portal vein were measured at 30 min after oral administration of maltose/miglitol to WT/HFD and WT/ND as previously reported [12]. Briefly, overnight-fasted mice were orally (10 μL/g BW) administered 2 g/kg maltose plus 10 mg/kg miglitol [kindly provided by Sanwa Kagaku Kenkyusho Co. Ltd. (Nagoya, Japan)] or vehicle (water). Thirty minutes after ingestion, the mice were anesthetized with isoflurane for 3 min and laparotomized to collect portal vein blood, which was subjected to measurement of plasma SCFAs, blood glucose, and plasma GIP. Blood glucose levels were measured as previously described [47]. Plasma levels of the SCFAs (acetate, propionate, butyrate, valerate, iso-butyrate, and iso-valerate) in the portal vein were measured as previously described [48].

### 4.3. Experiments of Lep^ob/ob^ Mice

#### 4.3.1. Fat Transplantation to *Lep^ob/ob^* Mice

Fat transplantation was performed as previously described [49]. Briefly, subcutaneous fat pads isolated from the abdominal walls of eight-week-old male WT/ND mice were transplanted into the subcutaneous area below the skin on the back of six-week-old *Lep^ob/ob^* mice. The fat pads isolated bilaterally from two donor mice were used for a single recipient. The sham operations were performed in *Lep^ob/ob^* mice and in WT mice.

#### 4.3.2. Oral Glucose Tolerance Test and Insulin Tolerance Test

The oral glucose tolerance test and insulin tolerance test were performed by oral gavage of 1 g/kg glucose and by intraperitoneal administration of 0.75 IU/kg human insulin to the mice fasted for 16 h. Blood glucose levels were measured at the time points indicated in the figures (Figure 6C,D).

### 4.4. Experiments of In Vivo GIP and IL-1Ra Secretion by LPS

Male wild-type mice were fasted overnight and subjected to measurement of plasma GIP and IL-1Ra levels in the portal vein at 3 h after intraperitoneal administration of 1 mg/kg LPS (Sigma-Aldrich, St. Louis, MO, USA). Portal vein blood was drawn in the same manner as was done in DIO mice (see above).

### 4.5. Experiments of In Vitro GIP Secretion from the Gut Organoids

Small intestinal organoid generation from mouse jejunum (section taken 8–13 cm from the gastric pylorus), maintenance, and two-dimensional (2D) culture were carried out as previously described [50]. GIP secretion experiments were performed using 2D organoid-derived cultures, 18–24 h after seeding cells onto 48-well plates. Cells were washed three times in warm saline buffer (138 mM NaCl, 4.5 mM KCl, 4.2 mM NaHCO_3_, 1.2 mM NaH_2_PO_4_, 2.6 mM CaCl_2_, 1.2 mM MgCl_2_, 10 mM HEPES; adjusted to pH 7.4 with 1 M NaOH, and supplemented on the day of experiment with 1 mM glucose and 0.1% fatty acid-free bovine serum albumin) before incubation in saline buffer for 30 min at 37 °C. The buffer was then completely removed before test reagents, dissolved in 150 µL saline buffer, were added to the organoid cultures. The test reagents used were 100 ng/mL LPS (Sigma-Aldrich, St. Louis, MO, USA), 100 µg/mL recombinant human IL-1Ra (PeproTech, Rocky Hill, NJ, USA), 10 ng/mL recombinant human MCP-1 (Peprotech), or 10 µM forskolin plus 10 µM 3-isobutyl-1-methylxanthine (IBMX) plus 10 mM glucose (all Sigma-Aldrich, St. Louis, MO, USA). After incubation for 2 h at 37 °C, supernatants were removed and centrifuged at 2000× *g* for 5 min at 4 °C, transferred to a fresh tube, and snap-frozen on dry ice.

### 4.6. ELISA Assay

For the measurements of GIP and IL-1Ra in the plasma, the blood sample was immediately mixed with EDTA (final 0.15% *w/v*). Concentrations of plasma and organoid secretion supernatant GIP and plasma IL-1Ra were measured using Rat/Mouse GIP (total) ELISA kit (Millipore, Billerica, MA, USA) and Mouse IL-1ra/IL-F3 Quantikine ELISA kit (R&D Systems, Inc. Minneapolis, MN, USA).

### 4.7. Quantitative Real-Time PCR

Total RNA was isolated from the gut (the duodenum and the terminal ileum) and the epididymal fat tissues and subjected to quantitative real-time PCR (qRT-PCR) analyses using Fast SYBR^®^ Green Master Mix (Applied Biosystems, Waltham, MA, USA), according to the manufacturer’s protocol. The primers used were 5′-gcgtcgtgattagcgatga-3′ and 5′-atggcctcccatctcctt-3′ for *Hprt*, 5′- acccttcaccaatgactcctatg -3′ and 5′- atgatgactgcagcaaatcgc -3′ for *Tbp*, 5′-atccgacaacaagacttcgt-3′ and 5′-atcatcactgaggctcttgg -3′ for *Gip*, 5′- cttccatccagttgccttcttg -3′ and 5′- aattaagcctccgacttgtgaag -3′ for *Il6*, 5′- ctttgaagttgacggacc -3′ and 5′- gaagctggatgctctcat -3′ for *Il1b*, 5′- cagccagatgcagttaacgc-3′ and 5′-gcctactcattgggatcatcttg-3′ for *Ccl2* (C-C motif ligand 2, MCP-1), 5′- cggagtccgggcaggt -3′ and 5′- gctgggtagagaatggatgaaca -3′ for *Tnfa*, 5′- aaggcagtggaagaccttgt -3′ and 5′- cttgcagggtcttttcccaga -3′ for *Ilira* (IL-1Ra).

### 4.8. Statistical Analysis

Results are expressed as means ± SEM. Differences between two groups were assessed using the unpaired two-tailed Student’s *t*-test unless otherwise noted. Datasets involving more than two groups were assessed by One-way ANOVA or Two-way ANOVA using GraphPad Prism, version 7.0 (GraphPad Software, San Diego, CA, USA). *p* < 0.05 was considered statistically significant.

## Abbreviations

GIP	glucose-dependent insulinotropic polypeptide
GLP-1	glucagon-like peptide 1
HFD	high-fat diet
ND	normal diet
DIO	diet-induced obesity
SCFA	short-chain fatty acid
IL-1Ra	interleukin-1 receptor antagonist
MCP-1	monocyte chemoattractant protein-1
IL-6	interleukin-6
TNF-α	tumor necrosis factor-α
IL-1β	interleukin-1β
IFN-β	interferon-β
LPS	lipopolysaccharide
PMA	phorbol myristate acetate
FABP5	fatty acid binding protein 5
α-GI	α-glucosidase inhibitor
PCoA	principal coordinate analysis
OTU	operational taxonomic unit
